# A Study on the Effectiveness of Spatial Filters on Thermal Image Pre-Processing and Correlation Technique for Quantifying Defect Size

**DOI:** 10.3390/s22228965

**Published:** 2022-11-19

**Authors:** Ho Jong Kim, Anuja Shrestha, Eliza Sapkota, Anwit Pokharel, Sarvesh Pandey, Cheol Sang Kim, Ranjit Shrestha

**Affiliations:** 1 Division of Mechanical Design Engineering, Chonbuk National University, Jeonju 561-756, Republic of Korea; 2 Gwangju Institute of Green Car Advancement, 55, Jingoksandanjungang-ro, Gwangsa-gu, Gwangju 62465, Republic of Korea; 3 Department of Mechanical Engineering, School of Engineering, Kathmandu University, Dhulikhel, Kavrepalanchok P.O. Box 6250, Nepal

**Keywords:** thermal imaging, pulsed thermography, noise, spatial filtering, signal to noise ratio

## Abstract

Thermal imaging plays a vital role in structural health monitoring of various materials and provides insight into the defect present due to aging, deterioration, and fault during construction. This study investigated the effectiveness of spatial filters during pre-processing of thermal images and a correlation technique in post-processing, as well as exploited its application in non-destructive testing and evaluation of defects in steel structures. Two linear filters (i.e., Gaussian and Window Averaging) and a non-linear filter (i.e., Median) were implemented during pre-processing of a pulsed thermography image sequence. The effectiveness of implemented filters was then assessed using signal to noise ratio as a quality metric. The result of pre-processing revealed that each implemented filter is capable of reducing impulse noise and producing high-quality images; additionally, when comparing the signal to noise ratio, the Gaussian filter dominated both Window Averaging and Median filters. Defect size was determined using a correlation technique on a sequence of pulsed thermography images that had been pre-processed with a Gaussian filter. Finally, it is concluded that the correlation technique could be applied to the fast measurement of defect size, even though the accuracy may depend on the detection limit of thermography and defect size to depth ratio.

## 1. Introduction

Image processing has now become a very highly demanded field of study and practice providing solutions to various real-life applications and is useful in many areas, disciplines and fields of art, and science and technology [1]. Due to the tremendous advancements achieved in microsystem technologies of infrared detectors, electronics, and computer science over the past few years, the application area of thermal imaging is growing fast in both science and technology [2]. Thermal imaging, also known as Infrared Thermography (IRT), is an optical measurement technique that deals with the acquisition and analysis of thermal data using a non-contact, high-speed thermal camera. Infrared detectors are the key to the IRT system, which detects the infrared radiation emitted by an object of interest and exploits Stefan–Boltzmann’s law to obtain temperature [3]. Every machine component and structure has a temperature range during normal operation. Defective machinery parts and components, faulty power supply and loose connections lead to abnormal temperature distribution and hence temperature is one of the best indicators of structural health [4]. IRT nowadays is not just limited to structural health monitoring but extends its application to R&D in various industries, including, nondestructive testing and evaluation (NDT&E), material characterization, manufacturing quality assurance, energy cost reduction, surveillance, night vision, agriculture, medical science and many more [5,6,7,8].

The basic concept of PT appeared in the 1960s when W.J. Parker et al. of the U.S. Naval Radiological Laboratory in California used the flash method to determine thermal diffusivity, heat capacity and thermal conductivity in a variety of materials [15]. Over the years, many scholars from around the world contributed to establishing a solid theoretical foundation and broadening its application areas. R. Monti and G. Mannara of the University of Naples, Italy inspected the complex honeycomb space structures and were able to detect the position of the defect and their size using PT [16]. V.P. Vavilov and R. Taylor of Tomsk Polytechnic University, Russia used PT for the NDT&E of bonded structures [17]. R.L. Thomas et al. of Wayne Institute University, USA used PT to detect corrosion and disbonding on the B737 testbed aircraft [18]. Xavier P.V. Maldague et al. of University Laval, Canada used PT for the NDT&E of carbon fiber reinforced plastic (CFRP) facings with aluminum honeycomb sandwich structures [19]. Xavier P.V. Maldague together with V.P. Vavilov inspected the defective aluminum specimen with internal and external corrosion, delaminations and water between the two sheets using PT [20]. Ranjit Shrestha of Kathmandu University, Nepal in collaboration with Wontae Kim of Kongju National University, South Korea and Stefano Sfarra of University of L’Aquila, Italy evaluated the ancient marquetry sample using PT [21]. Ranjit Shrestha and Wontae Kim evaluated the coating thickness [22,23] and also detected the wall thinning defects in steel structures [24] using PT. Fulvio Mercuri et al. from Università degli Studi di Roma Tor Vergata, Italy used PT to analyze repairs, decorative elements, and casting faults on bronzes, to detect texts hidden or damaged in ancient books/documents, and to characterize paint decorations [25]. Tomáš Kostroun and Milan Dvořák of Czech Technical University, Prague tested the adhesive joints of the wing spar caps made of a carbon composite using PT [26]. Ester D’Accardi et al. of Polytechnic University of Bari, Italy used PT to detect flaws in additive manufacturing components [27]. Many studies have been conducted recently on PT for defect detection, particularly on the estimation of defect depth followed by the improvement of signal to noise (SNR). C. Deemer et al. from Argonne National Laboratory, USA developed the experimental PT setup and discussed the peak difference time and peak slope time methods for determining the defect depth in continuous fiber ceramic composite components, concluding that peak-difference time depends on defect diameter, whereas peak-slope time appears to be constant with respect to diameter [28]. Xavier P.V. Maldague et al. of University Laval, Canada proposed pulse phase thermography (PPT) to investigate the high conductivity test samples with deeper defects with less influence of optical characteristics and surface infrared. It was also concluded that the experimental raw data is also affected by emissivity variations, reflections from the environment, non-uniform surface heating, and surface geometry sharp variations [11,13]. N. Rajic from Aeronautical and Maritime Research Laboratory, Australia used conventional flash thermography and proposed a singular value decomposition method based on principal component thermography (PCT) for NDT&E of delamination in the composite laminate test sample and demonstrated the evidence of a reduction in noise to a considerable extent with the high level of defect contrast [29]. S.M. Shepard et al. from Thermal Wave Imaging, Inc., USA proposed the thermal signal reconstruction (TSR) technique for the processing of experimental PT data and detect the flat bottom holes (FBH) defects in steel structure and concluded that the time derivative of the reconstructed data detects the defect with high sensitivity at earlier times with better SNR [30]. Yoonjae Chung et al. from Kongju National University, South Korea carried out an experimental investigation and compared the effectiveness of TSR, PPT and PCT methods in detecting the wall thinning defects in steel pipes. The results showed that TSR improved defect detectability, detecting the maximum number of defects, PPT provided the highest SNR, particularly for the deeper defects, and PCT provided the highest SNR for the shallower defects [24]. Nick Rothbart et al. of Federal Institute for Materials Research and Testing, Germany introduced a multi-parameter approach for calculating the POD for flash thermography with a large variation of test equipment as well as of tested materials and structures [31]. Haochen Liu from Cranfield University, UK proposed an automatic and accurate defect profile reconstruction method for PT using deep learning neural network and demonstrated its higher accurate and robust performance in sharp corners and edges of irregular defect profiles, which are commonly difficult for traditional processing [32].

**Figure 1:**
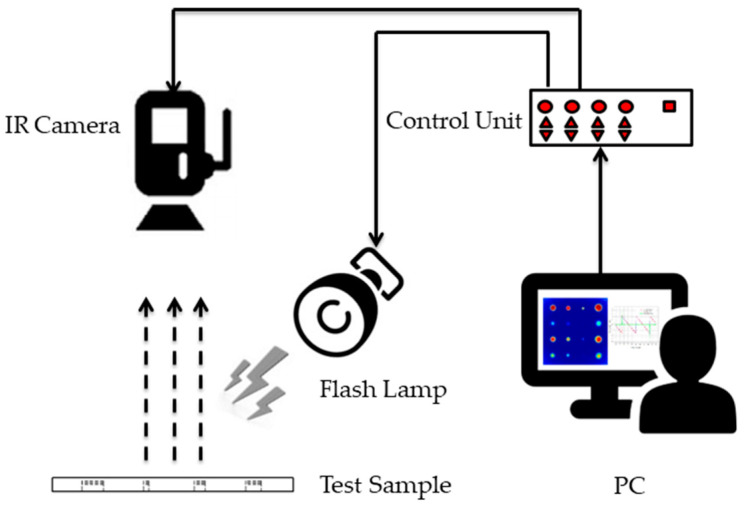
Schematic diagram of an experimental pulsed thermography inspection system.

Pulsed thermography (PT) is active thermography that integrates a thermal camera with flash heating. As shown in Figure 1, the PT consists of an IR Camera, Flash Lamps, Control Unit, Computer System, and a power supply. During the experiment, a short and high-power thermal energy is applied to the sample’s surface being examined, and the thermal response of the stimulated surface is observed in a transient state. The presence of any flaws alters the diffusion rate, causing defects to appear as surface regions of different temperatures when observing the surface temperature [9,10]. The detailed theory of PT can be found in the literature [11,12,13,14].

PT has been extensively studied for the estimation of defect depth, but it is also vital to note that the estimation of defect size has gotten comparatively little attention in the literature focusing only on threshold segmentation, edge extraction and temperature profile line approach to obtain the pixel value of the defective areas [33,34,35]. Lihua Yuan from Nanchang Hangkong University, China proposed maximum of standard deviation of sensitive region method to measure the FBH defect size in PVC plate by combining the threshold and edge extraction methods [36]. M.B. Saintey and D.P. Almond from University of Bath, UK employed the full width at half maximum contrast method to measure the size of the back drill hole [37]. Pengfei Zhu from Ningbo University, China introduced the temperature integral method for the quantitative analysis of the FBH defect size present in stainless steel plate and CFRP laminate [38]. Ranjit Shrestha et al. from Kongju National University employed the temperature line profile method to measure the size of FBH defect present in the stainless steel [39]. Ester D’Accardi et al. of the Polytechnic University of Bari, Italy, proposed a new empirical procedure for estimating FBHs and real defect size and depth in aluminum and glass fiber reinforced polymers by establishing a linear correlation between defect contrasts and relative aspect ratios [40]. Slawomir Grys of the Czestochowa University of Technology, Poland proposed relative incremental filtered contrast and filtered contrast methods for image segmentation and estimate the lateral dimensions of subsurface flaws in a polymethylmethacrylate slab [41]. Nevertheless, estimating the defect size is never easy because of the substantial noise that is present in the raw thermal data as a result of non-uniform heating and surface reflectivity. Additionally, there is always a chance that the edge information of the defect area may get blurred due to lateral temperature diffusion, making it difficult and very subjective to select the best image containing defect information.

The most important point to note is that despite extensive research on the transform domain for the post-processing of thermal images, the spatial domain for the pre-processing of raw thermal images has received relatively less attention. Raw thermal image sequence captured during experimentation, acquisition, and transmission are frequently corrupted by impulse noise, which can subsequently degrade the performance of post-processing algorithms. As a result, it is necessary to preserve the image detail by an effective noise removal method as a pre-processing step before using any subsequent image processing algorithm. This paper focused on the investigation of standard spatial domain filters during the pre-processing stage in the application for noise removal in thermal images. Additionally, the performance of selected spatial filters was evaluated using SNR metrics. Furthermore, the paper deals with the application of a correlation technique to estimate the defect size during the post-processing of thermal images.

The remaining section of the study is structured as follows: In Section 2, a brief overview of spatial filters, correlation technique, and signal to noise ratio is presented. The details of the test sample and experiment are presented in Section 3. The outcomes and analysis of each spatial filter and a correlation technique are covered in Section 4. Section 5 concludes by outlining the conclusions and prospective future research.

## 2. Background

### 2.1. Spatial Filters

Image denoising is a method of preserving image details, while removing as much random noise as possible from the image. The denoising methods are mainly divided into spatial domain and transform domain. Spatial domain filters operate the pixels on the raw images with high processing speed and are currently the standard pre-processing approach used before the post-processing of the image. The categories for spatial filters include linear (convolution) filters, such as Gaussian filter and Window Averaging filter and non-linear filters, such as Median filter. Through the years, traditional image processing has adopted the Gaussian filter, Window Average filter, and Median filter as standard spatial filters [42,43,44,45]

#### 2.1.1. Gaussian Filter

It is a linear smoothing filter that chooses the weights according to the shape of the Gaussian function. It is a kind of effective low-pass filter, especially to remove the noises that are subject to normal distribution. The Gaussian filter works by using the 2D distribution as a point-spread function. Gaussian kernel coefficients are sampled from the 2D Gaussian function and can be expressed by Equation (1) [46,47].

(1) $$G\left(x,y\right)=\frac{1}{2\pi \sigma^{2}}\;e^{\frac{x^{2}+y^{2}}{2\sigma^{2}}}$$

where σ is the standard deviation of the distribution. The distribution is assumed to have a mean of 0.

#### 2.1.2. Window Averaging Filter

It is a method of smoothing images by reducing the amount of intensity variation between neighboring pixels. It works by moving through the image pixel by pixel, replacing each value with the average value of the neighboring pixel, including itself. Large filters blur the image more. When the filter neighborhood straddles an edge, the filter will interpolate new values for pixels on the edge and so it will blur that edge. This may be a problem if sharp edges are required in the output [48].

#### 2.1.3. Median Filter

It is one of the well-known order statistic filters that is particularly effective at removing ‘salt and pepper’, ‘random’ and ‘Gaussian’ noise, while preserving edges. It works by moving through the image pixel by pixel, replacing each value with the median value of the neighboring pixel. The pattern of neighbors is called the “window”, which slides, pixel by pixel over the entire image pixel, over the entire image. The median is calculated by first sorting all the pixel values from the window into numerical order and then replacing the pixel being considered with the middle/median pixel value [49,50].

### 2.2. Correlation Technique

The correlation technique seeks to reveal similarities in the temporal behavior of temperature between a selected reference point and each image pixel. The correlation image will then display correlation coefficient values based on pixels. The correlation technique, which describes the similarity of two vectors, can be used to analyze a discrete signal with N sample points as an N-dimensional vector. The correlation coefficient for discrete signal x(n) and y(n), can be expressed by Equation (2) [51].

(2) $${Ρ}_{\mathrm{xy}}=\frac{\sum_{n=0}^{N-1}\left[x\left(n\right)-\mu_{x}\right]\left[y\left(n\right)-\mu_{y}\right]}{\sqrt{\sum_{n=0}^{N-1}{\left[x\left(n\right)-\mu_{x}\right]}^{2}\sum_{n=0}^{N-1}{\left[y\left(n\right)-\mu_{y}\right]}^{2}}}\;;\;-1\leq \rho_{\mathrm{xy}}\leq 1$$

where n designates the time point, $\mu_{x}$ and $\mu_{y}$ are the mean values of x(n) and y(n) respectively. It can be assumed that x(n) represents the sound area and y(n) represents the defective area.

Correlations involve two variables the first of which is the reference and the second one is the variable to be compared to the reference. It denotes the strength and direction of the linear relationship between a given temperature evolution reference and all of the temperature evolution of the pixels across the specimen under examination. As a result, it compiles the temporal information of a sequential image into a single one, similar to how the Fourier transform compiles the information of many images recorded over a given time period into a single resulting image.

### 2.3. Signal to Noise Ratio

Signal to noise ratio (SNR) is a measurement metric used to contrast two regions of interest (ROI). The SNR specifically determines the contrast value in a defective area and its neighborhood sound area in decibels [dB]. Two ROIs—one for the defective area and the other for the nearby sound area—are chosen for this purpose. ROI in the defective area will be regarded as “signal” (DROI), and ROI in the sound area will be regarded as “noise” (SROI). Equation (3) enables the calculation of the SNR [52,53].

(3) $$\mathrm{SNR}=20\log_{10}\left(\frac{\left|{\mathrm{DROI}}_{\mathrm{mean}}-{\mathrm{SROI}}_{\mathrm{mean}}\right|}{\sigma }\right)$$

where DROI_mean_ is the arithmetic mean of all the pixels inside the defective area; SROI_mean_ is the arithmetic mean of all the pixels inside the sound area and σ is the standard deviation of the pixels inside the sound region.

## 3. Methods and Materials

**Figure 2:**
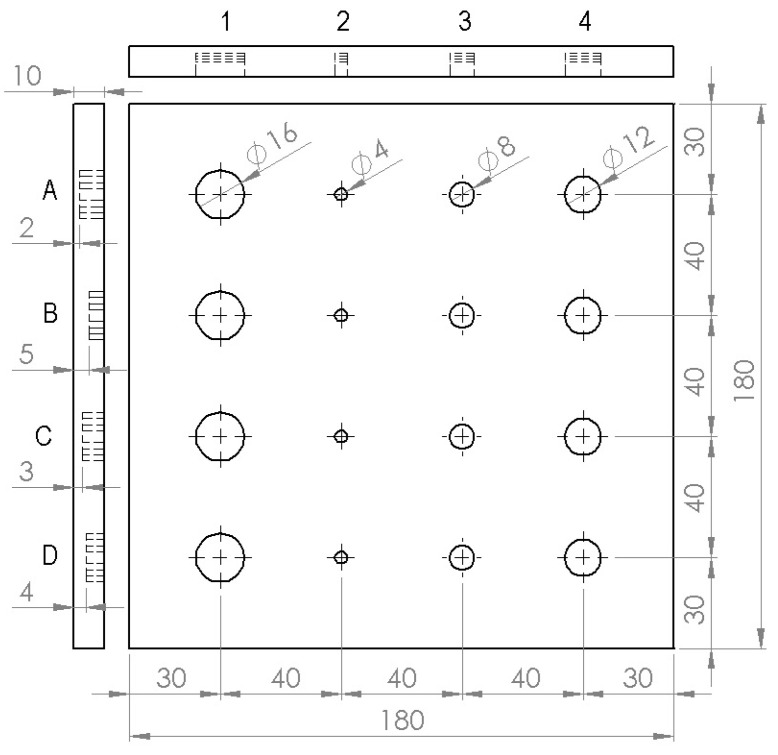
Schematic diagram of the test sample with geometry and position of artificial FBH defects of various sizes and depths [24].

In this study, the source images are selected from the article [24]. The source image includes the thermal data set where PT experimentation has been carried out over austenitic stainless steel (SUS 316) sample embedded with artificially simulated FBH of different sizes at varying depths. The sample’s schematic arrangement is depicted in Figure 2, where FBHs in each column represent defects that vary in depth from the top to the bottom row but have a constant diameter. Similar to this, each row’s FBHs depict defects that vary in size from left to right, while maintaining a constant depth. To create a consistent emissive surface, black KRYLON flat paint with an emissivity of 0.95 is coated on the sample’s front surface. The sample was then excited by a flash lamp of 6400 W-s, developed by BALCAR, France. The corresponding thermal response over the sample surface is captured with FLIR SC655 thermal camera operating at 7.5–13 μm spectral range with a maximum spatial resolution of 640 × 480 pixels, noise equivalent temperature difference (NETD) > 50 mk, spatial resolution (IFOV) 0.69 mrad, and accuracy ±2 °C. The frame rate of the thermal camera was fixed to 50 Hz to record 250 thermograms during the experimentation.

## 4. Results and Discussion

The total 250 thermal images with a spatial resolution of 640 × 480 pixels, acquired by FLIR R&D software during experimentation were imported to MATLAB^®^ R2020a. Even though FLIR R&D has inbuilt features for the pre-processing of thermal images, all preprocessing and post-processing were performed in MATLAB^®^ R2020a.

### 4.1. Selection of ROI and Pre-Processing with Spatial Filters

The raw thermal images not only consist of the test sample but also the area outside the sample (background) composed of highly noisy spectra and might hamper the good performance of the processing methods. Hence, ROI of size 480 × 480 pixels around the sample boundary is selected to remove the background information and a substantial saving of computing time. Then, the spatial filters previously described in Section 2.1 with kernel size 3 × 3 were implemented.

**Figure 3:**
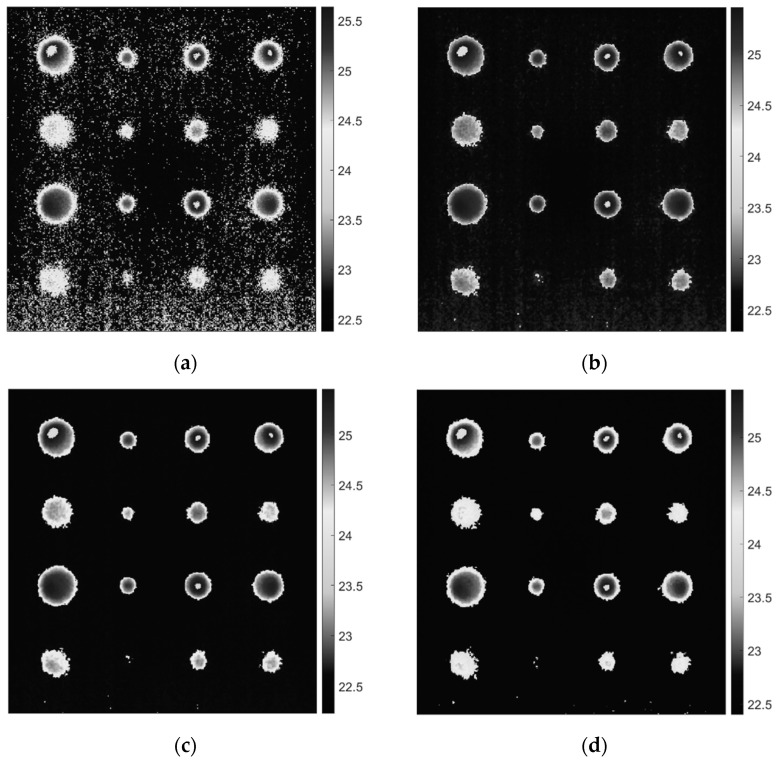
Comparison between the raw image and the filtered images, (**a**) Raw pulsed thermal image from the front surface at time 0.06 s [Frequency = 50 Hz, number of frames = 250, truncation window = 5 s], (**b**) Image after applying Gaussian filter of Kernel Size 3 × 3 and Sigma 0.85, (**c**) Image after applying Window Average filter of Kernel Size 3 × 3, and (**d**) Image after applying Median filter of Kernel Size 3 × 3.

**Figure 4:**
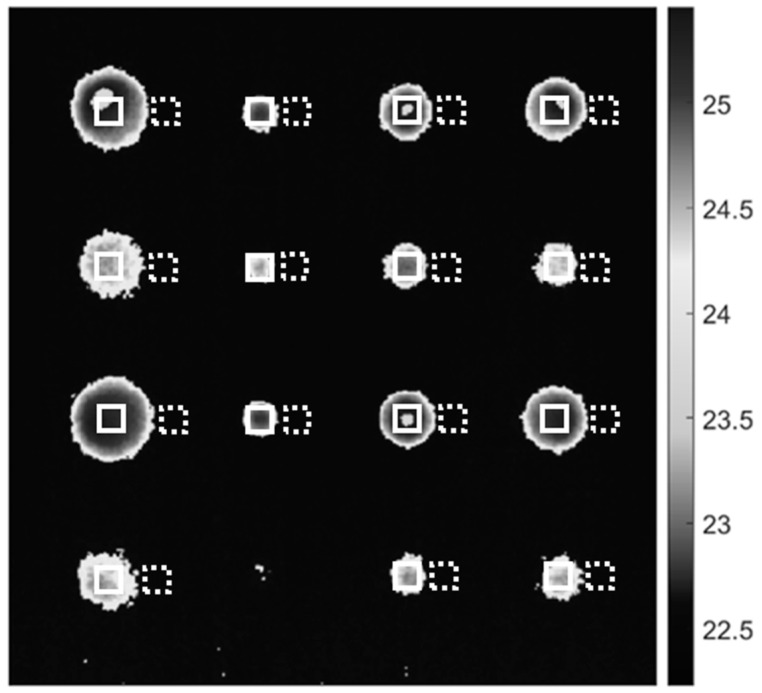
ROI representation for defective and sound areas for SNR computation. The red solid line enclosed area represents the defective areas, while the white dashed line enclosed area represents the sound area.

Figure 3 demonstrates the resultant images from the raw thermal image. Among 250 thermal images, the image at time 0.06 s was considered for the analysis because the maximum number of defects are detected with minimum noise. As illustrated in Figure 3, the filters produced high-quality, acceptable results. To assess the effectiveness of each implemented filter, the SNR of a particular defect is determined by calculating the ratio of the absolute difference between the mean of the defect area and adjacent sound area to the standard deviation of the sound area using Equation (3). For each defect, two ROIs of 6 × 6 pixels were considered, one in the center of the defect and one in the nearby sound area, as depicted in Figure 4. ROI in the defect area was regarded as ‘signal’ while in the sound area was regarded as ‘noise’.

Table 1 compares the SNR for each defect in raw and filtered images. As shown in Table 1, the Gaussian and Window Averaging filters improved the SNR of all 15 detected defects, whereas the Median filter improved the SNR of 12 defects, while maintaining the SNR of a single defect and decreasing the SNR of two defects. In addition, the Gaussian filter dominated the Window Averaging and Median filters in terms of SNR. For instance, in Table 1 for the shallowest and the largest defect A1; the raw thermal image had SNR of 39.65 dB; the Gaussian filtered image had SNR of 39.96 dB, which is an increment of 0.78%; Window Averaging filtered image had SNR of 39.95 dB, which is an increment of 0.76%; Median filtered image had SNR of 39.85 dB, which is an increment by 0.61%. Similarly, for the deepest and smallest defect B2, the raw thermal image had SNR of 38.29 dB; the Gaussian filtered image had SNR of 39.53 dB, which is an increment of 3.24%; Window Averaging filtered image had SNR of 39.04 dB, which is an increment of 1.96%; Median filtered image had SNR of 38.32 dB, which is an increment by 0.08%.

The most significant point to be made is that defects with Di indices are closer to surfaces than defects with Bi indices. This should lead to a higher SNR, according to the results so far. We suspected that this was due to the use of a single lamp, which was our limitation in this study, as well as non-uniform heating because the lamp was primarily focused on the center of the sample.

### 4.2. Post-Processing with a Correlation Technique

**Figure 5:**
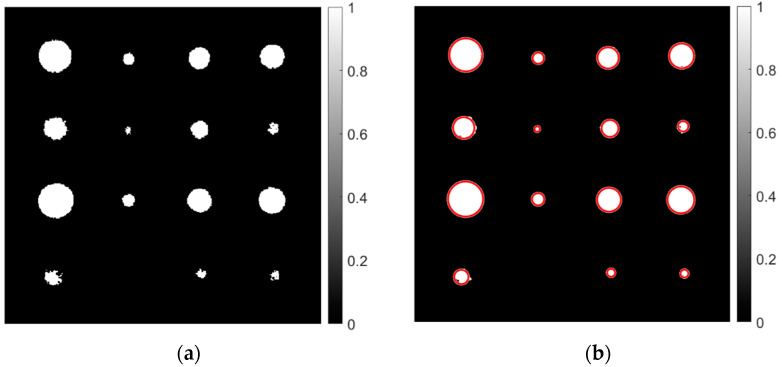
Resultant images using correlation technique, (**a**) Original correlation image and (**b**) correlation image with detected defect diameter.

The PT image sequence after pre-processing with Gaussian filter was then processed to obtain the correlation image. Figure 5a shows the correlation image obtained using Equation (2), where thermal profiles of each pixel have been cross-correlated with a reference profile and correlation coefficient contrast due to depth-dependent delay is used for defect detection. To measure the size of each defect, imfindcircles, a special function in MATLAB^®^ R2020a was used, which detects the diameter of defects as shown in Figure 5b and provides the defect size in pixel unit. Then, the mapping of pixel units in the actual unit was done through spatial calibration. Table 2 shows the errors in the estimation of defect size, while processing a PT image sequence using the correlation technique. As shown in Table 2, the error percentage varies with defect depth for the same defect size. It is also revealed that as the defect size to depth ratio increases, the error percentage decreases in the majority of the cases. For example, in Table 2, for the defect of the same size of 16 mm; defect A_1_ with a depth of 2 mm was detected with an error of 3.13%; defect C_3_ with a depth of 3 mm was detected with an error of 3.81%; defect D1 with a depth of 6 mm was detected with an error of 52.81%; and defect B_1_ with a depth 5 mm was detected with an error of 29.44%.

## 5. Conclusions and Future Works

In this study, a pulsed thermography image sequence aimed at detecting wall thinning defects in a steel structure was pre-processed with the three standard spatial filters before being processed with a correlation technique to determine the size of the defect. The presented results demonstrated that using spatial filters during the pre-processing stage can help to reduce impulse noise and to improve image quality. The performance evaluation of each spatial filter with respect to signal to noise ratio quality metric confirmed that the Gaussian filter is more effective than Window Average and Median filters. Furthermore, it is also confirmed that the correlation technique can be used to quickly estimate defect size, though the accuracy may be affected by the detection limit of pulsed thermography and defect size to depth ratio.

Future works will focus on the detection limit of pulsed thermography as well as the effects of defect depth on the estimation of defect size.

## Figures and Tables

**Table 1:** FBH defects and its corresponding SNR metric with respect to implemented filters.

Defect ID	SNR			
	Raw Image	Gaussian	Window Averaging	Median
**A_1_**	39.65	39.96	39.95	39.89
**A_2_**	39.58	40.23	40.04	39.67
**A_3_**	38.21	38.21	38.32	38.12
**A_4_**	39.87	40.56	40.52	39.91
**B_1_**	38.68	39.73	39.44	38.60
**B_2_**	38.29	39.53	39.04	38.32
**B_3_**	39.15	40.03	39.80	39.26
**B_4_**	38.21	39.46	38.92	38.21
**C_1_**	40.22	40.70	40.65	40.37
**C_2_**	39.87	40.44	40.25	39.89
**C_3_**	38.91	38.99	39.15	39.32
**C_4_**	40.29	40.74	40.65	40.33
**D_1_**	38.02	39.49	39.05	38.34
**D_2_**	-	-	-	-
**D_3_**	38.18	39.49	39.09	38.34
**D_4_**	37.91	39.49	39.06	38.40

Note: The symbol ‘-’ is the representation of non-detected defects.

**Table 2:** Estimation of defect size based on correlation technique and comparison to actual size.

Defect ID	Actual		Estimated		Error %
	Size (mm)	Depth (mm)	Size (Pixels)	Size (mm)	
**A_1_**	16	2	41.32	15.50	3.13
**A_2_**	4	2	10.72	4.02	0.50
**A_3_**	8	2	24.76	9.28	16.00
**A_4_**	12	2	29.70	11.14	7.17
**B_1_**	16	5	30.11	11.29	29.44
**B_2_**	4	5	7.64	2.87	28.25
**B_3_**	8	5	14.60	5.48	31.50
**B_4_**	12	5	23.65	8.87	26.08
**C_1_**	16	3	44.30	16.61	3.81
**C_2_**	4	3	12.08	4.53	13.25
**C_3_**	8	3	25.76	9.66	20.75
**C_4_**	12	3	32.71	12.27	2.25
**D_1_**	16	4	20.14	7.55	52.81
**D_2_**	4	4	-	-	-
**D_3_**	8	4	11.02	4.13	43.38
**D_4_**	12	4	11.20	4.20	65.00

Note: Symbol ‘-’ is the representation of non-detected defects.

## Data Availability

Not applicable.

## References

[1] Sarfraz M. (2020). Introductory Chapter: On Digital Image Processing. Digital Imaging.

[2] Vollmer M., Mllmann K. (2018). Infrared Thermal Imaging: Fundamentals, Research and Applications.

[3] Liu C., Cichon A., Królczyk G., Li Z. (2021). Technology Development and Commercial Applications of Industrial Fault Diagnosis System: A Review. Int. J. Adv. Manuf. Technol..

[4] Usamentiaga R., Venegas P., Guerediaga J., Vega L., Molleda J., Bulnes F. (2014). Infrared Thermography for Temperature Measurement and Non-Destructive Testing. Sensors.

[5] Lahiri B.B., Bagavathiappan S., Jayakumar T., Philip J. (2012). Medical Applications of Infrared Thermography: A Review. Infrared Phys. Technol..

[6] Maldague X.P. (2012). Nondestructive Evaluation of Materials by Infrared Thermography.

[7] Gade R., Moeslund T.B. (2014). Thermal Cameras and Applications: A Survey. Mach. Vis. Appl..

[8] Sarawade A.A., Charniya N.N. Infrared Thermography and its Applications: A Review. Proceedings of the 2018 3rd International Conference on Communication and Electronics Systems (ICCES).

[9] Carlomagno G.M., Meola C. (2002). Comparison between Thermographic Techniques for Frescoes NDT. NDT E Int..

[10] Theodorakeas P., Sfarra S., Ibarra-Castanedo C., Avdelidis N., Koui M., Maldague X., Ambrosini D., Paoletti D. The use of Pulsed Thermography for the Investigation of Art and Cultural Heritage Objects. Proceedings of the 5th International Conference on NDT of HSNT-IC MINDT.

[11] Maldague X., Marinetti S. (1996). Pulse Phase Infrared Thermography. J. Appl. Phys..

[12] Maldague X. (2001). Theory and Practice of Infrared Technology for Nondestructive Testing.

[13] Maldague X., Galmiche F., Ziadi A. (2002). Advances in Pulsed Phase Thermography. Infrared Phys. Technol..

[14] Sun J. (2006). Analysis of Pulsed Thermography Methods for Defect Depth Prediction. J. Heat Transfer..

[15] Parker W., Jenkins R., Butler C., Abbott G. (1961). Flash Method of Determining Thermal Diffusivity, Heat Capacity, and Thermal Conductivity. J. Appl. Phys..

[16] Monti R., Mannara G. Non Destructive Testing of Honeycomb Structures by Computerized Thermographic Systems(for Spacecraft Applications). Proceedings of the 34th International Astronautical Federation, International Astronautical Congress.

[17] Vavilov V., Taylor R. (1982). Theoretical and Practical Aspects of the Thermal Nondestructive Testing of Bonded Structures. Acad. Press Res. Tech. Nondestruct. Test..

[18] Favro L., Ahmed T., Han X., Wang L., Wang X., Kuo P., Thomas R. (1996). Thermal wave imaging of disbonding and corrosion on aircraft. Review of Progress in Quantitative Nondestructive Evaluation.

[19] Ibarra-Castanedo C., Piau J., Guilbert S., Avdelidis N.P., Genest M., Bendada A., Maldague X.P. (2009). Comparative Study of Active Thermography Techniques for the Nondestructive Evaluation of Honeycomb Structures. Res. Nondestr. Eval..

[20] Maldague X.P., Shiryaev V.V., Boisvert E., Vavilov V.P. Transient Thermal Nondestructive Testing (NDT) of Defects in Aluminum Panels. Proceedings of the Thermosense XVII: An International Conference on Thermal Sensing and Imaging Diagnostic Applications.

[21] Shrestha R., Sfarra S., Ridolfi S., Gargiulo G., Kim W. (2022). A Numerical–thermal–thermographic NDT Evaluation of an Ancient Marquetry Integrated with X-ray and XRF Surveys. J. Therm. Anal. Calorim..

[22] Shrestha R., Kim W. (2017). Evaluation of Coating Thickness by Thermal Wave Imaging: A Comparative Study of Pulsed and Lock-in Infrared thermography–Part I: Simulation. Infrared Phys. Technol..

[23] Shrestha R., Kim W. (2018). Evaluation of Coating Thickness by Thermal Wave Imaging: A Comparative Study of Pulsed and Lock-in Infrared thermography–Part II: Experimental Investigation. Infrared Phys. Technol..

[24] Chung Y., Shrestha R., Lee S., Kim W. (2020). Thermographic Inspection of Internal Defects in Steel Structures: Analysis of Signal Processing Techniques in Pulsed Thermography. Sensors.

[25] Mercuri F., Orazi N., Paoloni S., Cicero C., Zammit U. (2017). Pulsed Thermography Applied to the Study of Cultural Heritage. Appl. Sci..

[26] Kostroun T., Dvořák M. (2021). Application of the Pulse Infrared Thermography Method for Nondestructive Evaluation of Composite Aircraft Adhesive Joints. Materials.

[27] D’Accardi E., Krankenhagen R., Ulbricht A., Pelkner M., Pohl R., Palumbo D., Galietti U. (2022). Capability to Detect and Localize Typical Defects of Laser Powder Bed Fusion (L-PBF) Process: An Experimental Investigation with Different Non-Destructive Techniques. Prog. Addit. Manuf..

[28] Deemer C. Front-Flash Thermal Imaging Characterization of Continuous Fiber Ceramic Composites. Proceedings of the 23rd Annual Cocoa Beach Conference and Exposition.

[29] Rajic N. (2002). Principal Component Thermography for Flaw Contrast Enhancement and Flaw Depth Characterisation in Composite Structures. Compos. Struct..

[30] Shepard S.M., Lhota J.R., Rubadeux B.A., Wang D., Ahmed T. (2003). Reconstruction and Enhancement of Active Thermographic Image Sequences. Opt. Eng..

[31] Rothbart N., Maierhofer C., Goldammer M., Hohlstein F., Koch J., Kryukov I., Mahler G., Stotter B., Walle G., Oswald-Tranta B. (2017). Probability of Detection Analysis of Round Robin Test Results Performed by Flash Thermography. Quant. InfraRed Thermogr. J..

[32] Liu H., Li W., Yang L., Deng K., Zhao Y. (2022). Automatic Reconstruction of Irregular Shape Defects in Pulsed Thermography using Deep Learning Neural Network. Neural Comput. Appl..

[33] Wetsel Jr G.C., McDonald F.A. (1984). Resolution and Definition in Photothermal Imaging. J. Appl. Phys..

[34] Wang Z., Wan L., Xiong N., Zhu J., Ciampa F. (2021). Variational Level Set and Fuzzy Clustering for Enhanced Thermal Image Segmentation and Damage Assessment. NDT E Int..

[35] De Oliveira B.C., Nienheysen P., Baldo C.R., Gonçalves A.A., Schmitt R.H. (2020). Improved Impact Damage Characterisation in CFRP Samples using the Fusion of Optical Lock-in Thermography and Optical Square-Pulse Shearography Images. NDT E Int..

[36] Yuan L., Zhu X., Sun Q., Liu H., Yuen P., Liu Y. (2020). Automatic Extraction of Material Defect Size by Infrared Image Sequence. Appl. Sci..

[37] Saintey M., Almond D.P. (1995). Defect Sizing by Transient Thermography. II. A Numerical Treatment. J. Phys. D.

[38] Zhu P., Wu D., Yin L., Han W. (2022). Quantitative Detection of Defect Size Based on Infrared Thermography: Temperature Integral Method. Opt. Express.

[39] Ranjit S., Kang K., Kim W. (2015). Investigation of Lock-in Infrared Thermography for Evaluation of Subsurface Defects Size and Depth. Int. J. Precis. Eng. Manuf..

[40] D’Accardi E., Palumbo D., Galietti U. (2022). Experimental Procedure to Assess Depth and Size of Defects with Pulsed Thermography. J. Nondestr. Eval..

[41] Grys S. (2018). Determining the Dimension of Subsurface Defects by Active Infrared Thermography–experimental Research. J. Sens. Sens. Syst..

[42] Lin C., Kuo C., Lai C., Tsai M., Chang Y., Cheng H. (2011). A Novel Approach to Fast Noise Reduction of Infrared Image. Infrared Phys. Technol..

[43] Budzan S., Wyżgolik R. Noise Reduction in Thermal Images. Proceedings of the International Conference on Computer Vision and Graphics.

[44] Vardasca R., Gabriel J., Plassmann P., Ring F., Jones C. (2015). Comparison of Different Image Enhancing Techniques for Medical Thermal Images. J. Med. Imaging Health Inform..

[45] Alisha P., Sheela K.G. (2016). Image Denoising Techniques—An Overview. IOSR J. Electr. Commun. Eng..

[46] Wang M., Zheng S., Li X., Qin X. A New Image Denoising Method Based on Gaussian Filter. Proceedings of the International Conference on Information Science, Electronics and Electrical Engineering.

[47] Fan L., Zhang F., Fan H., Zhang C. (2019). Brief Review of Image Denoising Techniques. Vis. Comput. Ind. Biomed. Art.

[48] Ahmed R.J. (2011). Image Enhancement and Noise Removal by using New Spatial Filters. UPB Sci. Bull. Ser. C.

[49] Dinç İ., Dinç S., Sigdel M., Sigdel M.S., Aygün R.S., Pusey M.L. (2015). DT-Binarize: A decision tree based binarization for protein crystal images. Emerging Trends in Image Processing, Computer Vision and Pattern Recognition.

[50] Sagayam K.M., Bruntha P.M., Sridevi M., Sam M.R., Kose U., Deperlioglu O. (2021). A cognitive perception on content-based image retrieval using an advanced soft computing paradigm. Advanced Machine Vision Paradigms for Medical Image Analysis.

[51] Guo X., Vavilov V. (2015). Pulsed Thermographic Evaluation of Disbonds in the Insulation of Solid Rocket Motors made of Elastomers. Polym. Test..

[52] Shrestha R., Park J., Kim W. (2016). Application of Thermal Wave Imaging and Phase Shifting Method for Defect Detection in Stainless Steel. Infrared Phys. Technol..

[53] Shrestha R., Kim W. (2018). Non-Destructive Testing and Evaluation of Materials using Active Thermography and Enhancement of Signal to Noise Ratio through Data Fusion. Infrared Phys. Technol..

